# Differential Gemcitabine Sensitivity in Primary Human Pancreatic Cancer Cells and Paired Stellate Cells Is Driven by Heterogenous Drug Uptake and Processing

**DOI:** 10.3390/cancers12123628

**Published:** 2020-12-03

**Authors:** Manoj Amrutkar, Nils Tore Vethe, Caroline S. Verbeke, Monica Aasrum, Anette Vefferstad Finstadsveen, Petra Sántha, Ivar P. Gladhaug

**Affiliations:** 1 Department of Pharmacology, Institute of Clinical Medicine, University of Oslo, P.O. Box 1057 Blindern, 0316 Oslo, Norway; monica.aasrum@medisin.uio.no; 2 Department of Hepato-Pancreato-Biliary Surgery, Institute of Clinical Medicine, University of Oslo, P.O. Box 1171 Blindern, 0318 Oslo, Norway; i.p.gladhaug@medisin.uio.no; 3 Department of Pharmacology, Oslo University Hospital Rikshospitalet, P.O. Box 4950 Nydalen, 0424 Oslo, Norway; nvethe@ous-hf.no; 4 Department of Pathology, Oslo University Hospital Rikshospitalet, P.O. Box 4950 Nydalen, 0424 Oslo, Norway; c.s.verbeke@medisin.uio.no (C.S.V.); uxvene@ous-hf.no (A.V.F.); petra.santha@medisin.uio.no (P.S.); 5 Department of Pathology, Institute of Clinical Medicine, University of Oslo, P.O. Box 1072 Blindern, 0316 Oslo, Norway; 6 Department of Hepato-Pancreato-Biliary Surgery, Oslo University Hospital Rikshospitalet, P.O. Box 4950 Nydalen, 0424 Oslo, Norway

**Keywords:** human pancreatic ductal adenocarcinoma, primary cultures, pancreatic stellate cells and cancer cells, gemcitabine uptake and metabolism

## Abstract

**Simple Summary:**

Pancreatic ductal adenocarcinoma (PDAC, also known as pancreatic cancer) is one of the deadliest tumor types, characterized by poor prognosis, profound chemoresistance and overall low survival. Gemcitabine remains the standard of care for all stages of PDAC, however, with poor clinical benefits which is considered to be due to reduced drug availability in tumor cells. Gemcitabine-induced cytotoxicity depends upon sufficient drug uptake followed by intracellular activation. Pancreatic stellate cells (PSCs), a major stromal component of PDAC, were recently reported to scavenge active metabolites of gemcitabine, thereby making it unavailable for cancer cells. Gemcitabine uptake and processing in both tumor cells and PSCs, as well as expression analysis of its molecular metabolic regulators, was investigated in this study. We observed heterogeneous gemcitabine-induced cytotoxicity in different pancreatic cancer cells whereas it was absent in PSCs. The gemcitabine-induced cytotoxicity in pancreatic cancer cells was driven by differential expression of its molecular regulators.

**Abstract:**

Gemcitabine resistance in pancreatic ductal adenocarcinoma (PDAC) is attributed to cancer cell-intrinsic drug processing and the impact of the tumor microenvironment, especially pancreatic stellate cells (PSCs). This study uses human PDAC-derived paired primary cancer cells (PCCs) and PSCs from four different tumors, and the PDAC cell lines BxPC-3, Mia PaCa-2, and Panc-1, to assess the fate of gemcitabine by measuring its cellular uptake, cytotoxicity, and LC-MS/MS-based metabolite analysis. Expression analysis and siRNA-mediated knockdown of key regulators of gemcitabine (hENT1, CDA, DCK, NT5C1A) was performed. Compared to PSCs, both the paired primary PCCs and cancer cell lines showed gemcitabine-induced dose-dependent cytotoxicity, high uptake, as well as high and variable intracellular levels of gemcitabine metabolites. PSCs were gemcitabine-resistant and demonstrated significantly lower drug uptake, which was not influenced by co-culturing with their paired PCCs. Expression of key gemcitabine regulators was variable, but overall strong in the cancer cells and significantly lower or undetectable in PSCs. In cancer cells, hENT1 inhibition significantly downregulated gemcitabine uptake and cytotoxicity, whereas DCK knockdown reduced cytotoxicity. In conclusion, heterogeneity in gemcitabine processing among different pancreatic cancer cells and stellate cells results from the differential expression of molecular regulators which determines the effect of gemcitabine.

## 1. Introduction

Pancreatic ductal adenocarcinoma (PDAC), commonly known as pancreatic cancer, is characterized by a low rate of surgical resectability, profound chemoresistance, and overall 5-year survival of less than 7% [1,2]. For most patients, PDAC is a locally advanced or systemic disease at the time of diagnosis, thereby making chemotherapy a crucial component of the treatment [3]. Gemcitabine (2′,2′-difluoro-2′-deoxycytidine [dFdC]), a nucleoside analog, has long been the backbone of PDAC chemotherapy [4,5]. Although shown to be less effective than FOLFIRINOX (FOLinic acid, 5-FU, IRINotecan, and OXaliplatin) in the adjuvant setting [6], in metastatic PDAC gemcitabine remains the preferred drug in combination with nab-paclitaxel, or in monotherapy for patients not suitable for aggressive chemotherapy [7,8]. The poor clinical effect of gemcitabine has been considered to be due to its limited cellular uptake and impaired intracellular activation, causing overall low efficacy. However, the exact mechanisms underlying chemoresistance in PDAC remain elusive [9,10]. Therefore, a better understanding of pancreatic cancer biology, especially in the context of drug pharmacokinetics, is necessary.

Activated pancreatic stellate cells (PSCs, also referred to as cancer-associated fibroblasts-CAFs) are the major cellular component of PDAC, producing excessive amounts of various extracellular matrix (ECM) components [11,12,13]. The abundant deposition of ECM in the tumor stroma leads to vascular collapse with impaired drug delivery and acquired chemoresistance in PDAC [11,14]. Various secreted factors from stromal cells have recently been reported to promote gemcitabine resistance [15,16,17,18]. In addition, a recent study in murine pancreas suggests that PSCs/CAFs entrap the active form of gemcitabine intracellularly, thereby limiting its availability for cancer cells, and thus, reducing overall drug efficacy [19]. Several studies suggest that gemcitabine metabolism rather than the biophysical properties of the PDAC tissue matters most for chemoresistance to gemcitabine [9,20,21]. Following its cellular uptake in cancer cells, mainly by human equilibrative nucleoside transporter 1 (hENT1), gemcitabine undergoes a stepwise activation/phosphorylation process [9,22]. In the activation pathway, deoxycytidine kinase (DCK) catalyzes the initial step of phosphorylation by converting dFdC to gemcitabine monophosphate (dFdCMP) with subsequent generation of gemcitabine diphosphate (dFdCDP) and gemcitabine triphosphate (dFdCTP). dFdCTP is the main active metabolite of gemcitabine that exerts cytotoxic activity by inhibition of DNA replication [22]. The cellular fate of gemcitabine is also regulated by inactivation pathways. Cytidine deaminase (CDA) catalyzes the conversion of dFdC to 2′,2′-difluoro-2′-deoxyuridine (dFdU) [23]. In addition, dFdCMP is also inactivated by 5′-nucleotidase cytosolic 1A (NT5C1A), thereby limiting the generation of dFdCTP and causing chemoresistance [20]. Expression of these key regulators has been shown to correspond with the preclinical responses to gemcitabine and with patient survival [24,25,26,27].

Basic research in PDAC, including drug testing, has mainly been conducted using several commercially available cell lines [28]. However, their overall representativeness of the original tumor can be questioned, as various omics analyses have recently highlighted the presence of a high degree of heterogeneity of PDAC, both between individual tumors (inter-tumor heterogeneity) and also within the same tumor (intra-tumor heterogeneity) [29]. Moreover, some cells are derived from metastases, and all cell lines are prone to genetic drift. In attempts to overcome these challenges, various human PDAC-derived primary pancreatic carcinoma cell (PCC) lines have been established as experimental models for pancreatic cancer [30,31,32]. However, these models lack the complex input from stromal components found in all PDACs, particularly cues from the PSCs. Similarly, research on PSCs has mainly been conducted using a few commercially available PSC lines, which differ phenotypically and in their interactions with cancer cells compared to primary established PSCs, as shown in our recent study [33].

In a recent report, we demonstrated the establishment of a novel model system comprising human PDAC-derived paired primary cultures of primary cancer cells (PCCs), and PSCs derived from the same resected tumor specimen [34]. In the present study, paired primary cultures of PCCs and PSCs along with three commonly used PDAC cell lines, BxPC-3, Mia PaCa-2, and Panc-1, were used to explore the fate of gemcitabine in human PDAC. Cells were evaluated for gemcitabine uptake, intracellular metabolism, and cytotoxicity. Liquid chromatography tandem mass spectrometry (LC-MS/MS) was applied to quantify gemcitabine and its metabolites. Expression analysis of enzymes regulating gemcitabine metabolism was performed to understand the underlying mechanisms.

## 2. Results

### 2.1. Human PDAC-Derived Primary PCCs Are Chemosensitive Whereas PSCs Are Resistant to Gemcitabine

**Figure 1:**
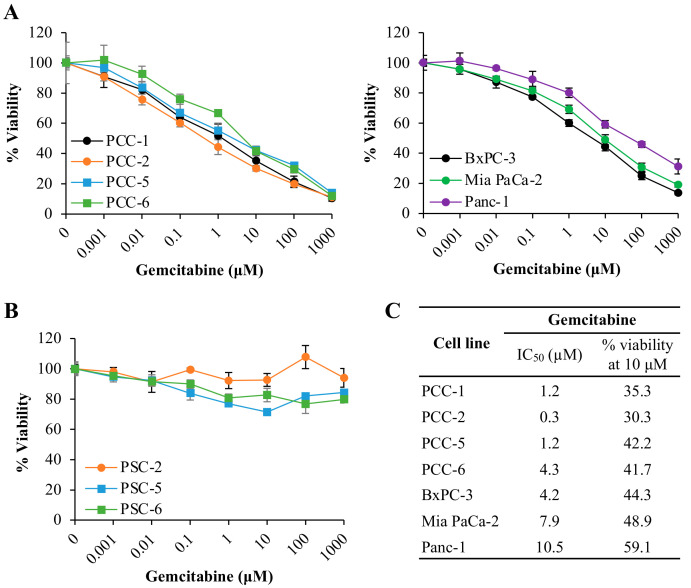
Assessment of gemcitabine-induced cytotoxicity. Human PDAC-derived primary cultures of PCCs, the PDAC cell lines (**A**), and the primary PSC cultures (**B**) seeded on 96-well plates at a density of 5000 cells/well were incubated with increasing concentrations of gemcitabine for 48 h, as indicated, and evaluated for cell viability using MTT assay. The absorbance signal corresponding to the formazan crystal formation in control cells was set to 100%, and subsequent gemcitabine-induced cytotoxicity was determined as a % change in the number of viable cells. (**C**) IC_50_ values for gemcitabine in respective PCC cultures and PDAC cell lines. Data are mean ± SEM of three replicates. Passage-4 PCCs and PSCs were used for chemosensitivity testing. PCC, pancreatic cancer cell; PSC, pancreatic stellate cell.

Primary cultures of PCCs and PSCs, as well as the PDAC cell lines BxPC-3, Mia PaCa-2, and Panc-1 cells, were examined for the cytotoxic effect of gemcitabine using the 3-(4,5-Dimethylthiazol-2-yl)-2,5-Diphenyltetrazolium Bromide (MTT) cell viability assay. Gemcitabine inhibited cell viability in all primary PCC cultures and the cell lines in a dose-dependent manner (Figure 1A), whereas none of the PSC cultures were sensitive to the cytotoxic effect of gemcitabine (Figure 1B). At a single concentration of gemcitabine (10 μM), cell viability was reduced by 58–70% in PCCs, and 40–56% in PDAC cell lines, respectively (Figure 1A,C). Notably, a significant cell population in each PCC culture, as well as in the cell lines, exhibited inherent resistance to gemcitabine. Of the total cell population, the proportion of cells with inherent resistance ranged from 11–14% among PCCs, and was 14, 19, and 31% among BxPC-3, Mia PaCa-2, and Panc-1 cells, respectively (Figure 1A). Furthermore, the primary PCCs were relatively more sensitive to gemcitabine than the three cell lines studied, as the IC_50_ values observed among the primary PCCs were overall significantly lower compared to those of the BxPC-3, Mia PaCa-2, and Panc-1 cells. A complete list of the IC_50_ values is provided in Figure 1C. Passage-6 PSCs stained with β-galactosidase for senescence detection revealed approximately 17–19% and 21–26% positive cells on Day 1 and Day 5, respectively (Figure S1A,B).

### 2.2. Gemcitabine Uptake Is Minimal in PSCs and Significantly Higher in PCCs

**Figure 2:**
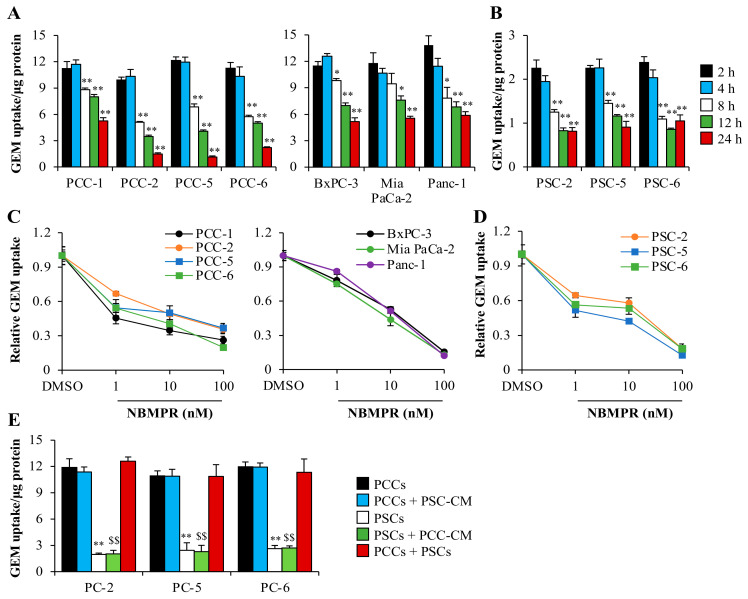
Assessment of intracellular gemcitabine transport. Human PDAC-derived primary PCCs (passage-4), the PDAC cell lines (**A**,**C**) and passage-5 primary PSCs (**B**,**D**) seeded in 96-well plates were incubated with [^3^H]-gemcitabine (50 nM) for indicated times (**A**,**B**) or for 4 h (**C**–**E**). (**C**,**D**) Cells incubated with hENT1 inhibitor NBMPR at indicated doses for 30 min prior exposure with [^3^H]-gemcitabine. (**E**) PCCs and PSCs co-cultured with conditioned medium from paired PSCs (PSC-CM) and PCCs (PCC-CM), or co-cultured together. Data are mean ± SEM of four replicates. (**A**,**B**) * *p* < 0.05, ** *p* < 0.01 for gemcitabine uptake in PCCs, PDAC cell lines and PSCs at 4, 8, 12 and 24 h compared to 2 h; (**E**) ** *p* < 0.01 and ^$$^ *p* < 0.01 for PCCs at basal vs. PSCs and PSCs + PCC-CM, respectively. GEM, gemcitabine; hENT1, human equilibrative nucleoside transporter 1; PC, pancreatic cancer; PCC, pancreatic cancer cell; PSC, pancreatic stellate cell.

Uptake of gemcitabine by the primary PCCs and the PDAC cell lines, as well as by the primary PSCs was assessed by measuring intracellular incorporation of radioactively labeled [^3^H]-gemcitabine for 24 h. In PCCs, the percentage of gemcitabine uptake ranged from 9.9–12.2% and 10.3–11.7% at 2 and 4 h, respectively (Figure 2A), whereas it was merely 2.2–2.4% and 1.9–2.3% in PSCs (Figure 2B). The percentage of gemcitabine uptake in BxPC-3 (11.5% and 12.6%) Mia PaCa-2 (11.8% and 10.7%), and Panc-1 (13.8% and 11.5%) cells was similar to that in the primary PCCs at 2 and 4 h, respectively (Figure 2A). Interestingly, the percentage gemcitabine uptake at 24 h was much lower in the primary PCCs (merely 1.2–5.2%) compared to the PDAC cell lines (5.2–5.9%; Figure 2A). None of the cell types displayed a statistically significant difference in gemcitabine uptake at 2 and 4 h, however, incubation for a longer duration revealed significantly reduced intracellular gemcitabine (Figure 2A,B). The equilibrative nucleoside transporter hENT1 is one of the key transporters of gemcitabine in PDAC [22,35]. Increasing doses of the hENT1-specific inhibitor S-(4-Nitrobenzyl)-6-thioinosine (NBMPR) dramatically decreased the intracellular levels of [^3^H]-gemcitabine in cancer cells (Figure 2C) and PSCs (Figure 2D). Interestingly, the intracellular levels of gemcitabine in PCCs were significantly higher at the highest dosage of NBMPR compared to those in PSCs (1.8-fold; Figure 2C,D), and PDAC cell lines (2.2-fold; Figure 2C,D).

In subsequent experiments, PCCs and PSCs cultured together (direct co-culture) or with conditioned medium (CM) obtained from their paired PSCs (PSC-CM) or PCCs (PCC-CM; indirect co-culture) were evaluated for gemcitabine transport into the cells (Figure 2E). No effect on gemcitabine transport capacity in PCCs or PSCs was observed following exposure to conditioned medium from their paired PSCs (PSC-CM) or PCCs (PCC-CM), respectively (Figure 2E). In all cultures studied, the overall gemcitabine transport in direct co-cultures was similar to that of individual cultures of PCCs only (Figure 2E).

### 2.3. High Variability in the Fate of Gemcitabine Following Its Uptake in Both PCCs and PSCs

**Figure 3:**
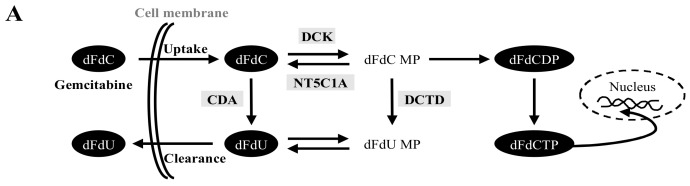

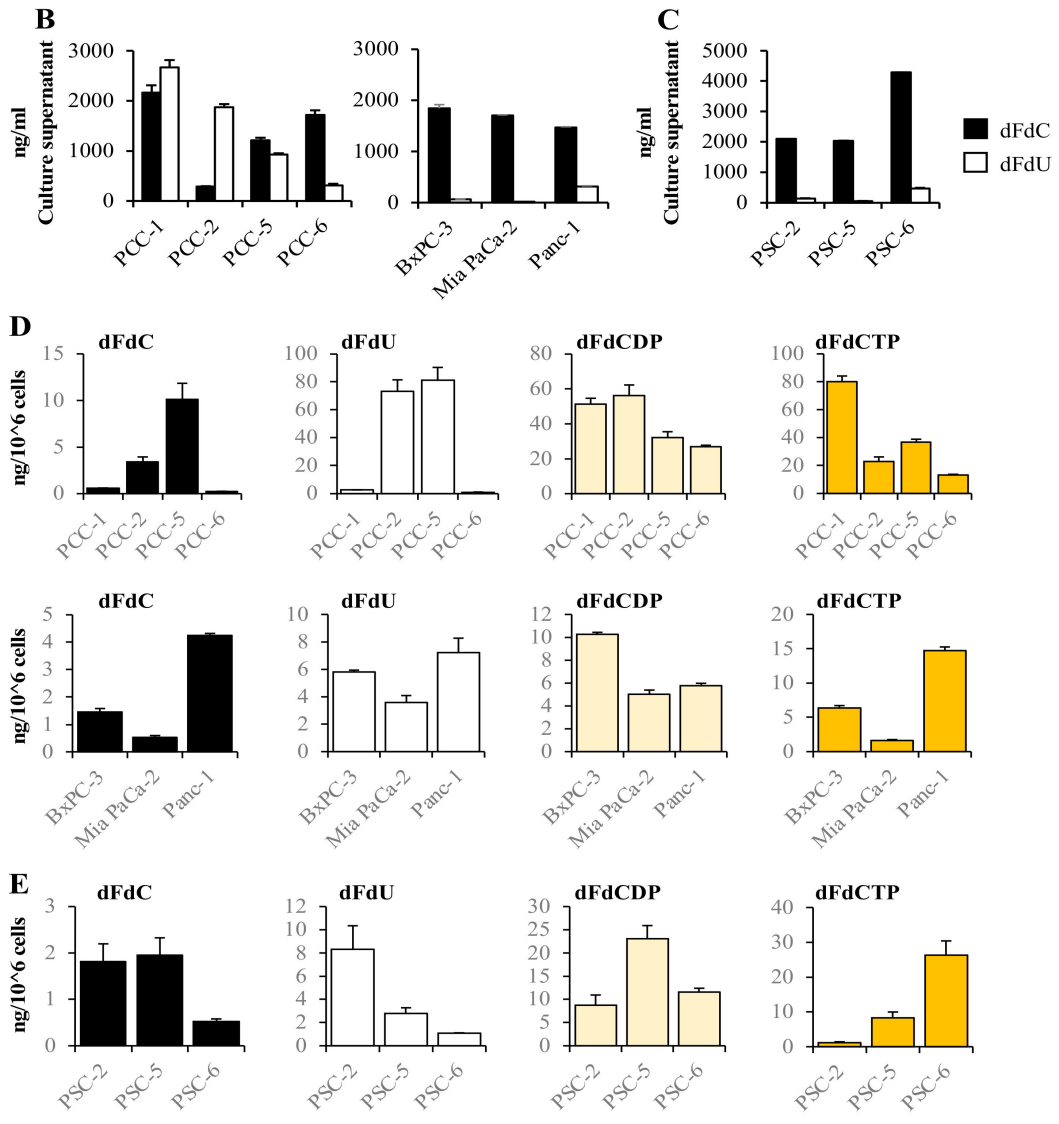
Pharmacokinetic profile of gemcitabine and its metabolites. (**A**) Schematic representation of intracellular activation of gemcitabine. (**B**–**E**) Individual cultures of human PDAC-derived primary PCCs (*n* = 4; passage-5), PDAC cell lines and PSCs (*n* = 3; passage-5) were incubated with gemcitabine (10 µM) for 2 h. Subsequently, culture supernatants (**B**,**C**) and cell pellets (**D**,**E**) were subjected to LC-MS/MS analysis. Extracellular amount of dFdC and dFdU measured in culture supernatants of PCCs and PDAC cell lines (**B**) and PSCs (**C**). Intracellular amount of gemcitabine prodrug dFdC, its inactive form dFdU, and its metabolites (dFdCDP, dFdCTP) in individual cultures of PCCs, PDAC cell lines (**D**), and PSCs (**E**). Data are mean ± SEM of three replicates. LC-MS/MS, liquid chromatography tandem mass spectrometry; PCC, pancreatic cancer cell; PSC, pancreatic stellate cell.

Following the transport into the cell, gemcitabine requires subsequent intracellular activation, i.e., enzymatic phosphorylation, to generate active molecules that exert the cytotoxic effects [22]. The process of intracellular activation of gemcitabine is presented schematically in Figure 3A. Cell pellets and culture supernatants were collected from each of the four PCC cultures, the PDAC cell lines BxPC-3, Mia PaCa-2, and Panc-1, and the three PSC cultures exposed to gemcitabine were subjected to LC-MS/MS analysis. The contents of gemcitabine prodrug dFdC and its inactivated form dFdU in individual cultures supernatants are provided in Figure 3B,C. The relative levels of dFdC and dFdU showed high variability among the various PCC-derived culture supernatants (Figure 3B), whereas the levels were relatively similar among the PSC-derived culture supernatants (Figure 3C). Moreover, there was a trend towards higher dFdC (2.1-fold) and lower dFdU (6.6-fold) in PSC-supernatants compared to supernatants from PCCs (Figure S2A). Analysis of gemcitabine metabolites in PCC and PSC pellets from the different individual source tumors revealed high inter-cellular and intra-tumor variability (Figure 3D,E). Similar intra-cellular variability was also seen between the three different PDAC cell lines (Figure 3D). Metabolite analysis of PSC pellets revealed lower intracellular concentrations of both dFdC (2.5-fold) and dFdU (9.7-fold) than in pellets from PCCs (Figure S2B). Similarly, average amounts of phosphorylated forms of gemcitabine dFdCDP and dFdCTP were 2.9- and 3.2-fold higher in PCCs than in PSCs, respectively (Figure S2B). The co-cultures of PCCs with their paired PSCs also showed high inter-tumor variability in both the culture supernatants (Figure S2C) and in the cell pellets (Figure S2D).

### 2.4. Expression Analysis of Key Determinants of Gemcitabine Uptake and Metabolism

**Figure 4:**
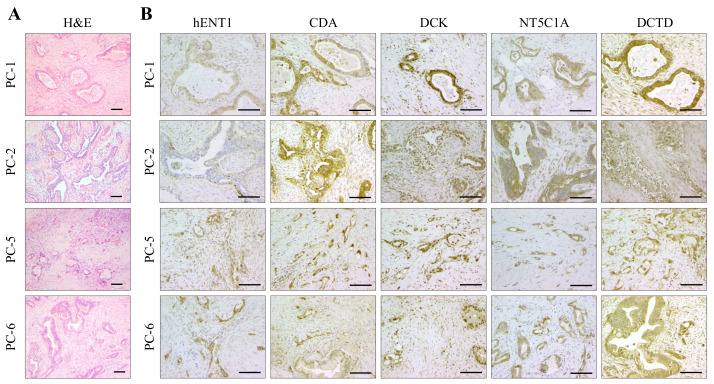
Morphology and expression analysis. Representative pictures from (**A**) H&E staining and (**B**) immunohistochemical staining of the source tumors from which the paired PCC and PSC cultures were obtained. Immunohistochemical pictures, showing expression of hENT1, CDA, DCK, NT5C1A, and DCTD in tumor cells. Stromal cells are occasionally weakly positive for hENT1, CDA, and NT5C1A, and up to moderately positive for DCK and DCTD. Some scattered leukocytes are positive for CDA and DCK. Scale bar = 50 µm. CDA, cytidine deaminase; DCK, deoxycytidine kinase; DCTD, deoxycytidylate deaminase; hENT1, human equilibrative nucleoside transporter 1; NT5C1A, 5′-nucleotidase cytosolic 1A; PC, pancreatic cancer.

Histopathological evaluation of H&E-stained sections of the source tumors (PC-1, -2, -5, and -6; PC: Pancreatic cancer) from which the paired PCCs and PSCs originated confirmed ductal adenocarcinoma in all four patients (Figure 4A). Immunohistochemical analysis of the source tumors revealed strong positive staining for hENT1, CDA, DCK, and NT5C1A in the cancer cells, whereas minimal expression was detected in the stromal cells (Figure 4B). DCTD was strongly expressed in both tumor cells and stromal cells (Figure 4B). Antibody details and the information regarding the controls are provided in Table S1.

**Figure 5:**
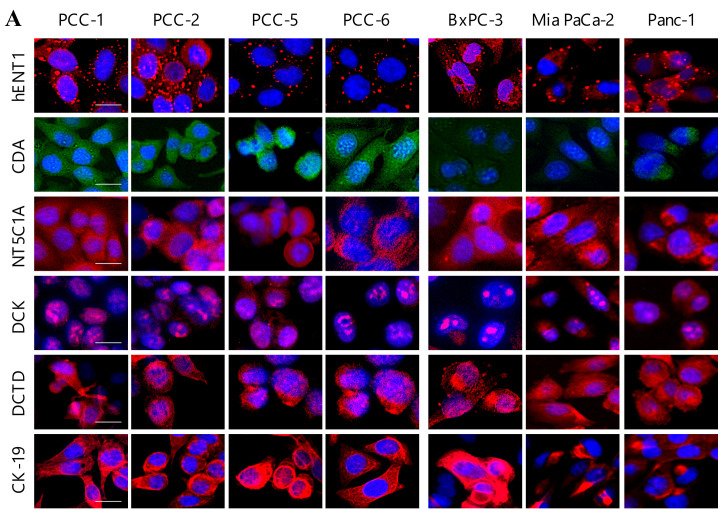

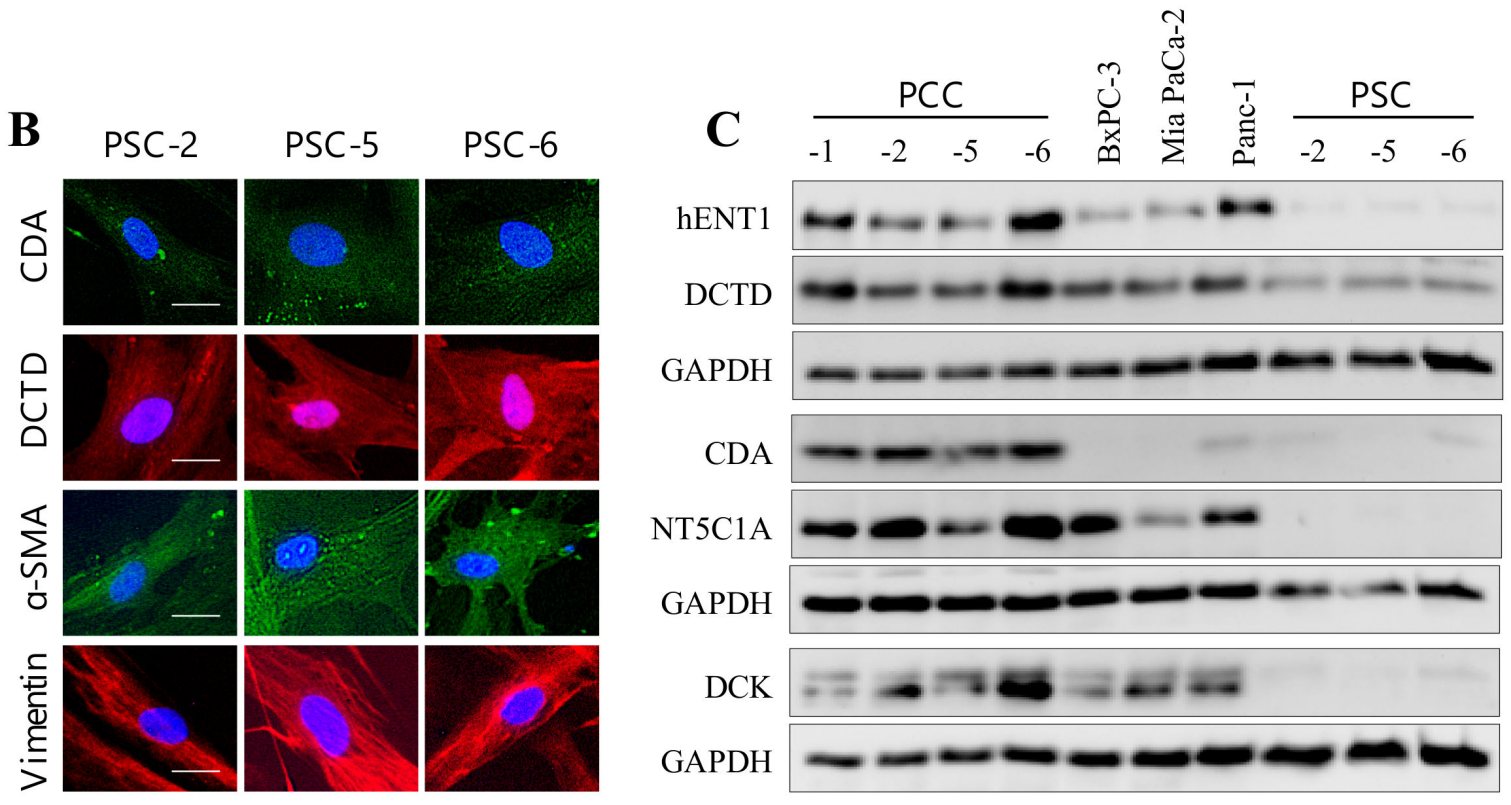
Expression analysis of key regulators of gemcitabine uptake and metabolism. Human PDAC-derived PCCs, the PDAC cell lines (**A**) and PSCs (**B**) seeded in 96-well plates were immunostained (**A**,**B**) or lysed and proteins, subjected to immunoblotting using antibodies against the indicated targets (**C**). Representative immunofluorescence images of three replicates of PCCs and cell lines (**A**) and PSCs (**B**). Scale bar = 10 μm. GAPDH was used as a loading control (**C**). PCCs (passage-4) and PSCs (passage-6) were used for western blot and immunofluorescence, respectively. CDA, cytidine deaminase; CK-19, cytokeratin 19; DCK, deoxycytidine kinase; DCTD, deoxycytidylate deaminase; hENT1, human equilibrative nucleoside transporter 1; NT5C1A, 5′-nucleotidase cytosolic 1A; PCC, pancreatic cancer cell; PSC, pancreatic stellate cell; α-SMA, α-smooth muscle actin.

Expression analysis of the key enzymes that regulate gemcitabine uptake (hENT1) and intracellular metabolism (CDA, DCK, NT5C1A, and DCTD) was performed in cancer cells and PSCs by immunofluorescence (Figure 5A,B) and by western blot analysis using total cell lysates (Figure 5C). The analysis revealed a variable hENT1 expression among both cancer cells and PSCs (Figure 5A–C). Notably, a significantly lower hENT1, CDA, DCK, NT5C1A, and DCTD expression was observed in all PSCs compared to PCCs (Figure 5C). Compared to PCCs, PDAC cell lines showed a significantly lower CDA expression (Figure 5A,C), whereas NT5C1A expression was variable among PCCs and lower in PDAC cell lines (Figure 5A,C). Moreover, western blot analysis of total cell lysates from cancer cells and PSCs exposed to gemcitabine (10 µM) for 48 h revealed no change in the expression of hENT1, CDA, DCK, NT5C1A, and DCTD as compared to non-exposed cells (Figure S3), suggesting that gemcitabine did not affect the expression of these enzymes in either cell type studied. Of note, all PCCs cultures and PDAC cell lines showed expression of the epithelial marker cytokeratin 19 (CK-19; Figure 5A), whereas all PSC cultures demonstrated a strong expression of mesenchymal marker vimentin and the stellate cell activation marker α-smooth muscle actin (αSMA; Figure 5B).

### 2.5. Gemcitabine-Induced Cytotoxicity in Pancreatic Cancer Cells Is Primarily Determined by hENT1 and DCK Expression

**Figure 6:**
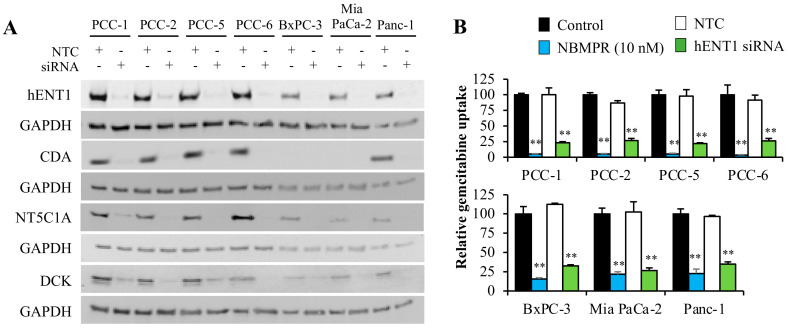

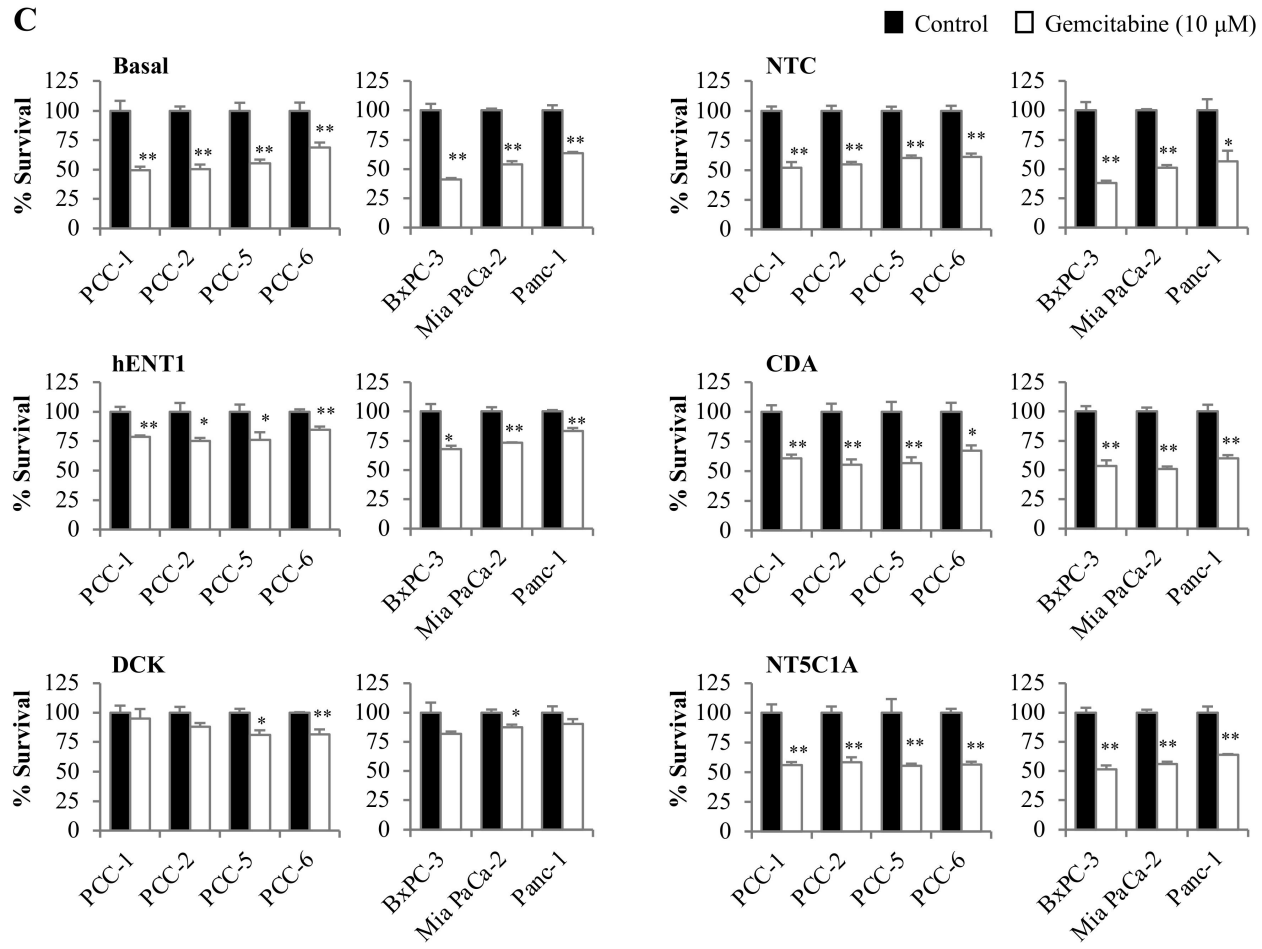
Gemcitabine transport and cytotoxicity assessment following inhibition of key regulators of gemcitabine uptake and metabolism. Human PDAC-derived PCCs (passage-7) and PDAC cell lines were transiently transfected with negative control siRNA (NTC) or siRNA against hENT1, CDA, DCK, or NT5C1A. (**A**) Cells incubated for 72 h post-transfection were lysed and proteins, subjected to immunoblotting using antibodies against the indicated targets. GAPDH was used as a loading control. (**B**) Gemcitabine uptake in cells pre-incubated with hENT1 inhibitor NBMPR or transfected with NTC or siRNA against hENT1, followed by exposure to [^3^H]-gemcitabine (50 nM) for 4 h. ** *p* < 0.01 for control vs. NBMPR or NTC vs. hENT1 siRNA. (**C**) Non-transfected (Basal) cells and cells transfected for the indicated targets were exposed to gemcitabine (10 µM) for 48 h and assessed for MTT-based cell viability. * *p* < 0.05, ** *p* < 0.01 for control vs. gemcitabine. Data are mean ± SEM of four replicates (**B**,**C**). CDA, cytidine deaminase; DCK, deoxycytidine kinase; hENT1, human equilibrative nucleoside transporter 1; NTC, negative transfection control; NT5C1A, 5′-nucleotidase cytosolic 1A; PCC, pancreatic cancer cell.

Cancer cells were transiently transfected with negative transfection control (NTC) or siRNA against hENT1, CDA, DCK, and NT5C1A (siRNA details are provided in Table S2). The successful knockdown was validated by western blot analysis (Figure 6A). Transfection efficiency was observed to be between 55.3–73.3%, based on GAPDH abundance (Figure S4A), and between 64.0–75.4%, based on siGLO immunofluorescence (Figure S4B). Compared to NTC, a significantly lower gemcitabine uptake was observed in all cancer cells transfected with hENT1 siRNA (Figure 6B). However, the degree of reduction was significantly smaller in transfected cells than in non-transfected cells that were pre-exposed to NBMPR compared with their respective controls (Figure 6B). Notably, this difference was lower in the PDAC cell lines than in PCCs (Figure 6B).

Non-transfected cancer cells displayed a varying degree of gemcitabine-induced reduction in cell viability, with highest reduction in cell viability in PCC-1 (51%) and BxPC-3 (58%; Figure 6C). Similar responses were also seen in PCCs transfected with NTC. Average gemcitabine-induced reduction in cell survival in PCCs was 44% and 43%, and in cell lines, 47% and 52% in non-transfected cells and NTC cells, respectively (Figure 6C). Furthermore, cells transfected with siRNA against hENT1 and DCK revealed a substantial reduction of the gemcitabine-induced decrease in cell survival, whereas knockdown of CDA and NT5C1A did not result in significant changes in gemcitabine-induced cytotoxicity compared to non-transfected and NTC (Figure 6C). The average gemcitabine-induced reduction in cell survival of PCCs was merely 21% and 14% following knockdown of hENT1 and DCK, respectively, whereas the average was 25% and 14% in the cell lines (Figure 6C).

### 2.6. Correlation between Gemcitabine Chemosensitivity and Its Intracellular Metabolites or Expression of Key Regulator Proteins

**Figure 7:**
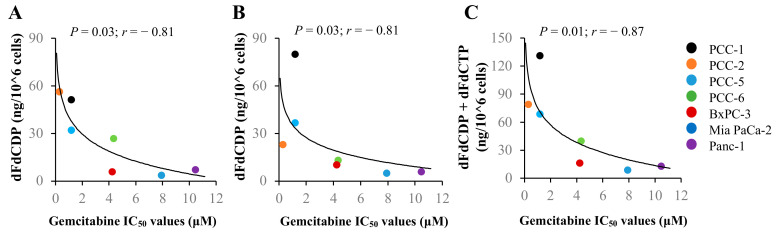
Assessment of the correlation between gemcitabine sensitivity and levels of its active metabolites. Correlation plots of gemcitabine IC_50_ values vs. levels of (**A**) dFdCDP, (**B**) dFdCTP, and (**C**) dFdCDP + dFdCTP. IC_50_ values for gemcitabine were calculated using GraphPad Prism 6 software. Non-linear logarithmic regression was applied for the variable vs. IC_50_ values and were analyzed by the Spearman correlation test. dFdC, 2′,2′-difluoro-2′-deoxycytidine (gemcitabine); dFdCDP, gemcitabine diphosphate; dFdCTP, gemcitabine triphosphate; PCC, pancreatic cancer cell.

To assess whether or not the combined effects of activation and inactivation of gemcitabine correlated with chemosensitivity, we first assessed the correlation between gemcitabine IC_50_ values and the level of the different intracellular gemcitabine metabolites in the cancer cells (Figure 7A–C, Table S3). Individual levels of dFdCDP (Figure 7A), dFdCTP (Figure 7B), or combined, dFdCDP + dFdCTP (Figure 7C), each showed a negative correlation with the gemcitabine IC_50_ values. Furthermore, we explored the correlations between gemcitabine IC_50_ values and the protein expression of its key regulators normalized to GAPDH in the cancer cells (adapted from [36,37]). Gemcitabine IC_50_ values did not significantly correlate with the individual relative expressions of hENT1, DCK, CDA, NT5C1A, or DCTD (Table S3). Interestingly, an inverse correlation was observed between gemcitabine sensitivity in terms of IC_50_ values and the ratio of hENT1 to CDA (*p* < 0.05) and the ratio of hENT1 × DCK to CDA × DCTD (*p* < 0.05; Table S3). A significant correlation was not seen between gemcitabine IC_50_ values and the ratio of hENT1 × DCK to CDA × NT5C1A × DCTD (Table S3). Moreover, a trend toward positive correlation was seen between gemcitabine IC_50_ values and the ratio of hENT1 × DCK to CDA (*p* = 0.07; Table S3). None of the ratio hENT × DCK, CDA × NT5C1A, hENT1/CDA × NT5C1A, or DCK/CDA × NT5C1A correlated with gemcitabine sensitivity (Table S3).

## 3. Discussion

Since the report by Burris et al. in 1997 [4], gemcitabine has been considered as first-line therapy for locally advanced and metastatic PDAC, despite only marginal survival benefits. Clinical failure of PDAC treatment with gemcitabine has been partly attributed to impaired drug delivery to the stroma-rich tumor microenvironment and to chemoresistance (both inherent and acquired) [9,11]. Stromal depletion strategies aiming at enhanced drug delivery to PDAC have been explored during the past decade, but have generally failed to generate significant clinical benefits [38]. Moreover, enhanced drug delivery does not ensure that the chemotherapeutic agent is metabolically available and active against malignant cells [9,22]. Thus, reassessing the mechanisms of gemcitabine processing and activation in the cells that comprise a PDAC tumor is critical for improved therapy.

In the present study, human PDAC-derived paired primary PCC and PSC cultures, and the PDAC cell lines BxPC-3, Mia PaCa-2, and Panc-1 were used to explore the cellular fate of gemcitabine. All PCCs and PDAC cell lines exhibited a dose-dependent reduction in cell viability following exposure to gemcitabine. Interestingly, PCCs demonstrated overall higher chemosensitivity than the PDAC cell lines. In contrast, PSCs showed resistance to gemcitabine regardless of the dose used, which is in line with our previous findings [15]. Of note, the MTT assay was used in this study to determine gemcitabine-induced cytotoxicity in vitro over a short time period (48 h). Among various cytotoxicity testing assays, the MTT assay is unquestionably well-characterized and reliable, and is, therefore, used routinely in both academia and industry for cytotoxicity testing. However, there are certain limitations with respect to the interpretation of this assay. For example, the MTT assay is a short-term assay, where a reduction in the MTT signal is a surrogate for cell killing. It does not determine whether the surviving cells display a temporarily reduced growth rate, or even enter a transient growth arrest during treatment. Despite these limitations, the MTT assay is the most practical and feasible cytotoxicity assay currently available.

Transport into the cell is a rate-limiting step that determines the fate of gemcitabine. Overall, cancer cells displayed a 5-fold higher uptake of gemcitabine compared to PSCs, indicating that PSC’s resistance to gemcitabine is most likely related to impaired drug uptake. Gemcitabine transport across the cell membrane is regulated by various nucleoside transporters, and kinetic studies have shown that hENT1 is the key transporter of gemcitabine in PDAC [9,22,35]. Expression analysis revealed a significantly lower hENT1 expression in PSCs as compared to cancer cells, supporting the observed lower gemcitabine uptake by PSCs. Moreover, the dose-dependent reduction in gemcitabine transport following exposure of cancer cells and PSCs to the hENT1 inhibitor NBMPR, as well as the reduced gemcitabine transport following the knockdown of hENT1 in cancer cells, support the importance of hENT1 as the prime cellular transporter of gemcitabine in both cell types.

Intracellular availability of gemcitabine alone is not sufficient to generate the desired cytotoxic effects, because the latter is also dependent on the intracellular activation of gemcitabine to generate its active metabolite dFdCTP along with an appropriate balance between intracellular activation and deactivation [9,39]. LC-MS/MS analysis revealed a significantly lower intracellular amount of active gemcitabine metabolites, dFdCDP and dFdCTP, in the PSCs than in the PCCs, which can be explained by significantly lower expression of hENT1 and DCK in PSCs as compared to PCCs. Notably, despite similar average intracellular dFdC levels, the metabolites dFdCDP and dFdCTP were significantly lower in PDAC cell lines compared to PCCs. In addition, lower levels of active metabolites of gemcitabine were also reflected by lower gemcitabine sensitivity in PDAC cell lines compared to PCCs. DCK is a main rate-limiting enzyme in intracellular gemcitabine activation following its uptake. The observed lower active metabolites of gemcitabine in PDAC cell lines compared to PCCs may indicate reduced enzymatic activity of DCK in the PDAC cell lines. DCK activity has been reported to correlate with gemcitabine sensitivity in cancer cells [40]. DCK activity could be affected by genetic variants or interacting proteins [41,42].

CDA inactivates gemcitabine by converting dFdC to dFdU, and its role in in vivo gemcitabine pharmacokinetics and in vitro drug sensitivity is well described. It is only recently that the role of CDA in intracellular gemcitabine metabolism in PDAC cells has been examined in a quantitative manner [23]. It was observed that the concentrations of dFdU and dFdCTP differed considerably between BxPC-3, Mia PaCa-2, and Panc-1 cells, depending on the activity of CDA [6]. Similarly, in the present study, a considerable variation in the expression of CDA was observed among both PDAC cell lines and PSCs, and it was overall significantly lower in both PSCs and PDAC cell lines than in PCCs. LC-MS/MS analysis revealed significantly lower levels of dFdU in PSCs and PDAC cell lines than in PCCs, which can be explained by a significantly lower CDA expression observed in PSCs and cell lines. Expression analysis of NT5C1A in PCCs further confirmed its strong expression in cancer cells of resected PDACs, as indicated in a recent study by Patzak et al. [20]. However, our findings do not support the role of NT5C1A in mediating gemcitabine resistance, because levels of NT5C1A expression were similar in PDAC cell lines and PCCs, while chemosensitivity was significantly lower in PDAC cell lines. Of note, NT5C1A expression in PSCs was below detectable levels. Furthermore, the key gemcitabine regulators hENT1, DCK, CDA, and NT5C1A were expressed to varying degrees among the different cancer cells, whereas PSCs displayed overall significantly lower expression than the cancer cells. In addition, a similar heterogeneity was also observed regarding gemcitabine uptake, cytotoxicity, and in the overall gemcitabine metabolite profile. Taken together, these observations support the observed differences in pharmacokinetic profiles of gemcitabine between PCCs and PSCs, as well as between the various cancer cells. Of note, although the sample size is too small to compare, no clear differences were observed between PCCs derived from treatment-naïve (PCC-1 and PCC-2), and neoadjuvantly treated (PCC-5 and PCC-6) PDACs.

Modulation of cellular enzymes regulating transport and metabolism (i.e., hENT1, DCK, CDA, and NT5C1A) may influence the cytotoxic effect of gemcitabine. In cancer cells, gemcitabine-induced cytotoxicity was significantly lower following knockdown of hENT1 and DCK, whereas knockdown of CDA and NT5C1A had no impact, highlighting the importance of a balanced expression of the key regulators of gemcitabine metabolism for treatment effects to occur. In an effort to characterize the relationship between gemcitabine cytotoxicity and its uptake and processing in a quantitative manner, the protein expression of its key regulators in the cancer cells was calculated. Correlations between gemcitabine IC_50_ values and gemcitabine metabolites or protein expression of gemcitabine metabolism markers were investigated in the total cohort of cancer cells (PCCs and PDAC cell lines combined). Although merging two different groups of malignant cells originating from the same cancer may not be optimal, the analysis revealed a clear pattern. Individual levels of dFdCDP, dFdCTP, or combined, dFdCDP+dFdCTP, each showed a negative correlation with the gemcitabine IC_50_ values. Furthermore, there was a strong correlation between gemcitabine IC_50_ values and the ratios hENT1/CDA and hENT1 × DCK/CDA × DCTD, however in the opposite direction. This finding could be at least partially explained by a significantly lower CDA expression in PDAC cell lines compared to PCCs. Of note, individual protein expression of hENT1, DCK, CDA, DCTD, or NT5C1A showed no correlation with gemcitabine sensitivity. In addition, none of the other ratios, including hENT × DCK, CDA × NT5C1A, hENT1/CDA × NT5C1A, or DCK/CDA × NT5C1A, correlated with gemcitabine sensitivity.

A recent study by Hessmann et al. reported that gemcitabine was more effectively accumulated in fibroblast-rich primary tumors as compared to the less stoma-rich liver metastases in a murine PDAC model [19]. It further suggested that drug scavenging and entrapment of large amounts of dFdCTP by fibroblasts results in a reduced availability of gemcitabine for tumor cells, thereby reducing drug efficacy. Our results show that human PDAC-derived primary PSCs accumulate very little gemcitabine, indicating that in human PDAC, PSCs may not have a prominent drug scavenging role. We have no obvious explanation for these divergent results other than the species, genetic, and biological differences between genetically engineered mouse tumor models and human tumors [43,44]. We cannot entirely exclude the possibility that the PCCs and PSCs might have acquired new properties as a consequence of the cell isolation and culture processes, thus making them behave differently from the cells in situ in the tumor tissue, although similar isolation and culturing techniques were also employed in the Hessmann study [19], making this explanation less likely. It should also be noted that primary human PSCs differ in many functional characteristics from their transformed murine counterparts [33]. In addition, different subtypes of PSCs/CAFs have been reported in terms of divergent tumor-promoting effects and therapy resistance [45,46,47,48]. The existence of subtypes of PSCs and possibly different proportions of such subtypes with distinct gemcitabine metabolic capacities in individual PDAC tumors might further complicate this picture.

## 4. Material and Methods

### 4.1. Reagents

Reagents were purchased from the following sources: Dulbecco’s modified Eagle’s medium containing 4.5 g/L glucose (DMEM), penicillin-streptomycin (Pen-Strep), Amphotericin B, Trypsin/EDTA, fetal bovine serum (FBS), and PierceTM BCA protein assay kit from Thermo Fisher Scientific (Waltham, MA, USA); bovine serum albumin (BSA), gemcitabine hydrochloride, phosphate-buffered saline (PBS), 3-(4,5-Dimethylthiazol-2-yl)-2,5-Diphenyltetrazolium Bromide (MTT), and S-(4-Nitrobenzyl)-6-thioinosine (NBMPR) from Sigma-Aldrich (St. Louis, MO, USA); [^3^H]-gemcitabine from Moravek Biochemicals Inc. (Brea, CA, USA); Ultima Gold from Perkin Elmer (Waltham, MA, USA).

### 4.2. Cell Culture and Patient Information

Human PDAC-derived paired primary cultures of cancer cells (PCCs) and stellate cells (PSCs) were obtained from the same surgically resected specimens by using the outgrowth method [34,49]. Four paired primary cultures were grown from different PDACs, of which two were treatment-naïve (PC-1, PC-2), and two were neoadjuvantly treated (PC-5, PC-6) with one cycle of Folfirinox followed by five cycles Gemzar-Abraxane (PC-5), and seven cycles Gemzar (PC-6), respectively. Detailed information on the establishment and characterization of cells, clinicopathological features of the source tumors, patient survival, and treatment is provided in our recent study [34]. The established cultures were designated as PCC- and PSC-1, -2, -5, and -6, respectively. Of note, PSCs from PC-1 were of the insufficient amount to perform further experiments, thus, PSC-1 cells were not included in the study. The PSC cultures were evaluated for senescence using Senescence Cells Histochemical Staining Kit (Sigma-Aldrich). PCC and PSC cultures between passage-3 and -8, as well as the PDAC cell lines BxPC-3, Mia PaCa-2, and Panc-1 purchased from American Type Culture Collection (ATCC, Manassas, VA, USA) were cultured and maintained in DMEM supplemented with 10% FBS, 1% Pen-Strep and 1% Amphotericin B.

The study protocol and patient consent documents were approved by the Regional Committee for Medical and Health Research Ethics (REC South East, project number 2015/738) and followed the Helsinki Declaration. Written informed consent was obtained from the patients whose tumor tissue was used for the study.

### 4.3. Chemosensitivity

PCCs, PDAC cell lines, and PSCs seeded in 96-well plates at a density of ~5000 cells/well were treated for 48 h with gemcitabine (concentration range of 0.001–1000 μM). To determine gemcitabine-induced cytotoxicity, the metabolic activity reflecting cell viability in response to varying concentrations of gemcitabine and the corresponding IC_50_ values were determined using the MTT assay [15]. In principle, in the MTT assay, the extent of formazan crystal formation is determined by the absorbance measurements, which correlates with the number of viable cells. IC_50_ is the amount of drug required for inhibition of cancer cell growth by 50% compared to untreated controls.

### 4.4. Gemcitabine Uptake

PCCs, PDAC cell lines, and PSCs cultured to confluence in 96-well plates were incubated with transport buffer containing 50 nM [^3^H]-gemcitabine at 37 °C for indicated times (2–24 h) and assessed for gemcitabine uptake as described previously [15]. In further gemcitabine uptake experiments, the co-culture system of paired PCCs and PSCs was used, as described in our previous study [15]. Briefly, for indirect co-culture experiments, PCCs and PSCs seeded individually at a density of ~5000 cells/well in 96-well plates were exposed to conditioned medium from their paired PSCs (PSC-CM) and PCCs (PCC-CM), respectively, for 48 h. Conditioned medium was prepared as described previously [34]. For direct co-cultures, paired PCCs and PSCs were seeded at an equal density of ~3000 cells/well in 96-well plates and cultured together for 48 h. The co-cultures were exposed to [^3^H]-gemcitabine for 4 h and evaluated for intracellular uptake of gemcitabine. When indicated, cells were pre-incubated for 30 min with the nucleoside inhibitor NBMPR (1–100 nM), or DMSO (0.1%). Subsequently, cells were lysed using 0.2 M NaOH and submitted to a liquid scintillation counter to determine cell-associated radioactivity in counts per minute (CPM). Protein concentration was determined using the BCA protein assay kit. CPM values were normalized to protein concentrations.

### 4.5. Western Blot Analysis

Whole cell lysates were prepared using Laemmli buffer, and protein aliquots were separated by electrophoresis (SDS-PAGE) as described previously [15,33]. The proteins were transferred using a semi-dry transfer system (Bio-Rad, Hercules, CA, USA), blocked in 5% non-fat dry milk, and incubated overnight at 4 °C with the primary antibodies as indicated. Blots were then incubated with HRP-conjugated secondary antibodies at room temperature for 1 h and visualized with LumiGLO^®^ (KPL, Gaithersburg, MD, USA). Densitometric analyses were performed using Labworks Software (UVP, Cambridge, UK); data were normalized to GAPDH. Antibody details and the information regarding the controls are provided in Table S1. Uncropped images of western blots are provided in Figure S5.

### 4.6. LC-MS/MS Analysis

PCCs, PDAC cell lines, and PSCs grown in 12-well plates were incubated for 2 h with culture media containing gemcitabine (10 µM). Subsequently, cell pellets and culture supernatants were collected and subjected to quantitative LC-MS/MS analysis of gemcitabine prodrug dFdC and its active metabolites gemcitabine diphosphate (dFdCDP), gemcitabine triphosphate (dFdCTP), and the inactivated form dFdU. The assay was designed as described previously with some modifications [19,50]. Additional details are provided in Supplementary Materials and methods.

### 4.7. Histology, Immunohistochemistry and Immunofluorescence

PDAC tissues were fixed in 10% neutral buffered formalin for 24 h and transferred to 70% ethanol. Tissues were embedded in paraffin, and 3–5 μm serial sections were processed for hematoxylin and eosin (H&E) staining or for immunohistochemical staining using standard protocols as previously described [19]. Briefly, tissue sections were incubated with primary antibodies at 4 °C overnight, followed by incubation with secondary antibodies as listed in Table S1.

For immunofluorescence, cells cultured in 96-well plates were fixed in 4% formaldehyde, followed by overnight incubation with various primary antibodies and subsequent incubation with Alexa Fluor-conjugated secondary antibodies. DAPI was used for nuclear staining. Images were captured using EVOS FLoid Cell Imaging Station (Thermo Fisher Scientific). Antibody details are provided in Table S1.

### 4.8. Transient Transfections (RNAi Interference)

PCCs and PDAC cell lines were transfected with siRNAs targeting hENT1, CDA, DCK, or NT5C1A (Thermo Fisher Scientific) or non-targeting scrambled siRNA (AM4611; Thermo Fisher Scientific) using Lipofectamine RNAiMAX reagent (Invitrogen, Waltham, MA, USA). Transfection efficiency was assessed using Select GAPDH control siRNA (Thermo Fisher Scientific; 4390849) based GAPDH abundance and siGLO Green Transfection Indicator (Dharmacon, Lafayette, CO, USA; D-001630–01) based nuclear immunofluorescence staining. siRNA details are provided in Table S2. Cells were incubated for 72 h post-transfection in serum-free medium before being exposed to [^3^H]-gemcitabine for assessment of gemcitabine transport. For assessment of gemcitabine-induced cytotoxicity, transfected cells were incubated with gemcitabine (10 µM) for 48 h, followed by MTT-based cell viability assessment.

### 4.9. Statistical Analysis

All values are expressed as mean ± standard error of mean (SEM). Statistical analysis was performed using GraphPad Prism 6 (GraphPad Software Inc., San Diego, CA, USA). Statistical differences between groups were assessed with an unpaired two-tailed Student’s *t* test, with a value of *p* < 0.05 considered statistically significant. Correlations between gemcitabine sensitivity and levels of gemcitabine metabolites or protein expression of its metabolic markers were analyzed by the Spearman correlation test.

## 5. Conclusions

Human PDAC-derived primary PCCs and PDAC cell lines demonstrate dose-dependent gemcitabine sensitivity, whereas the primary PSCs are chemoresistant. These findings are supported by higher uptake and intracellular activation of gemcitabine in the cancer cells compared to PSCs. The study revealed a novel positive correlation between gemcitabine sensitivity and levels of its active metabolites or protein expression ratios hENT1/CDA and hENT1 × DCK/CDA × DCTD in cancer cells. Notably, considerable inter-cellular heterogeneity in cancer cells was also observed in gemcitabine uptake, processing, and expression of its metabolic regulators. Lastly, this study suggests that human PDAC-derived PSCs are less able to accumulate, and mediate intracellular activation of gemcitabine compared to PCCs.

## Supplementary Materials

The following are available online at https://www.mdpi.com/2072-6694/12/12/3628/s1, Materials and methods: Quantification of gemcitabine and its metabolites, Table S1: Antibodies details, Table S2: siRNA details, Table S3: Gemcitabine IC50 values and its metabolites levels or protein expression of metabolizing enzymes in pancreatic cancer cells, Figure S1: Senescence-associated β-galactosidase staining, Figure S2: Pharmacokinetic profile of gemcitabine and its metabolites, Figure S3: Expression of key regulators of gemcitabine metabolism was not influenced by gemcitabine exposure, Figure S4: Transfection efficiency, Figure S5: Uncropped western blot images.

## Abbreviations

CDA	Cytidine deaminase
DCK	Deoxycytidine kinase
DCTD	Deoxycytidylate deaminase
dFdC	2′,2′-Difluoro deoxycytidine
dFdCDP	2′,2′-Difluoro deoxycytidine- 5′-diphosphate
dFdCTP	2′,2′-Difluoro deoxycytidine-5′-triphosphate
dFdU	2′,2′-Difluorodeoxyuridine
hENT1	Human equilibrative nucleoside transporter 1
LC-MS/MS	Liquid chromatography tandem mass spectrometry
MTT	3-(4,5-Dimethylthiazol-2-yl)-2,5-diphenyltetrazolium bromide
NBMPR	S-(4-Nitrobenzyl)-6-thioinosine
NTC	Negative transfection control
NT5C1A	5′-Nucleotidase cytosolic 1A
PC	Pancreatic cancer
PCC	Pancreatic cancer cell
PDAC	Pancreatic ductal adenocarcinoma
PSC	Pancreatic stellate cell
PSC-CM	PSC-conditioned medium
TME	Tumor microenvironment
