# Supplementary material for: Differential Gemcitabine Sensitivity in Primary Human Pancreatic Cancer Cells and Paired Stellate Cells Is Driven by Heterogenous Drug Uptake and Processing
Source: Cancers (Basel). 2020 Dec 3;12(12):3628. doi: 10.3390/cancers12123628 (PMC7761836; doi:10.3390/cancers12123628)
Supplement: Supplementary file 1 [file cancers-12-03628-s001.pdf]

Differential Gemcitabine Sensitivity in Primary Human Pancreatic Cancer Cells and Paired Stellate Cells is Driven by Heterogenous Drug Uptake and Processing

Manoj Amrutkar, Nils Tore Vethe, Caroline S. Verbeke, Monica Aasrum, Anette Vefferstad Finstadsveen, Petra Sántha and Ivar P. Gladhaug

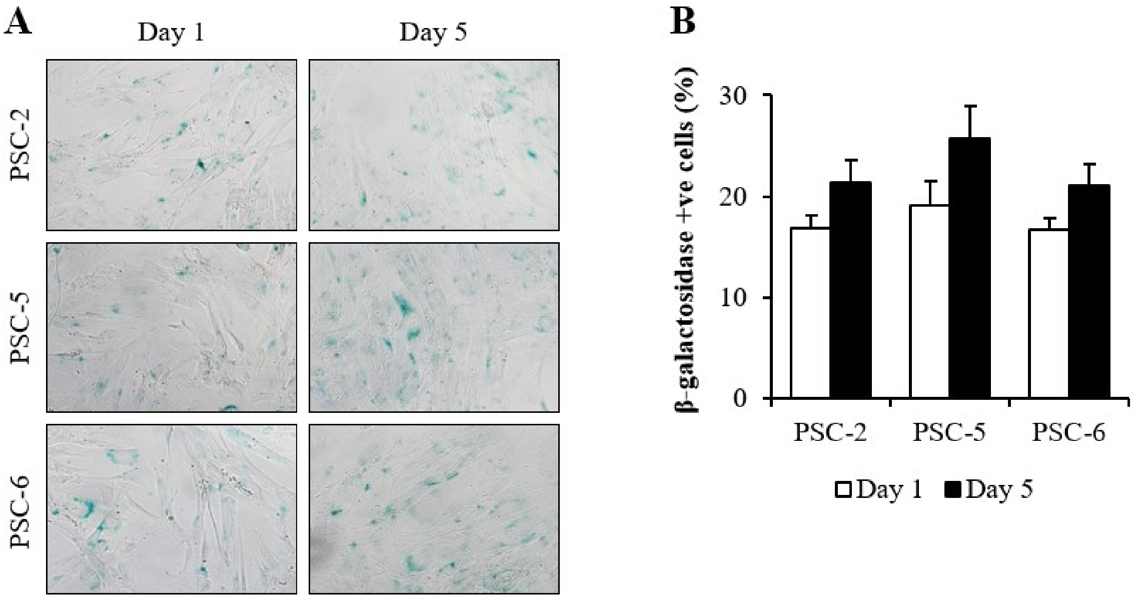

Figure S1. Senescence-associated β-galactosidase staining. (**A**) Human PDAC-derived primary cultures of PSCs seeded on 96-well plates were stained for β-galactosidase at 24 h (Day 1) and 120 h (Day 5) after cell seeding and (**B**) percentage positive cells were counted using ImageJ software. Data are mean ± SEM of three replicates. PSC, pancreatic stellate cell.

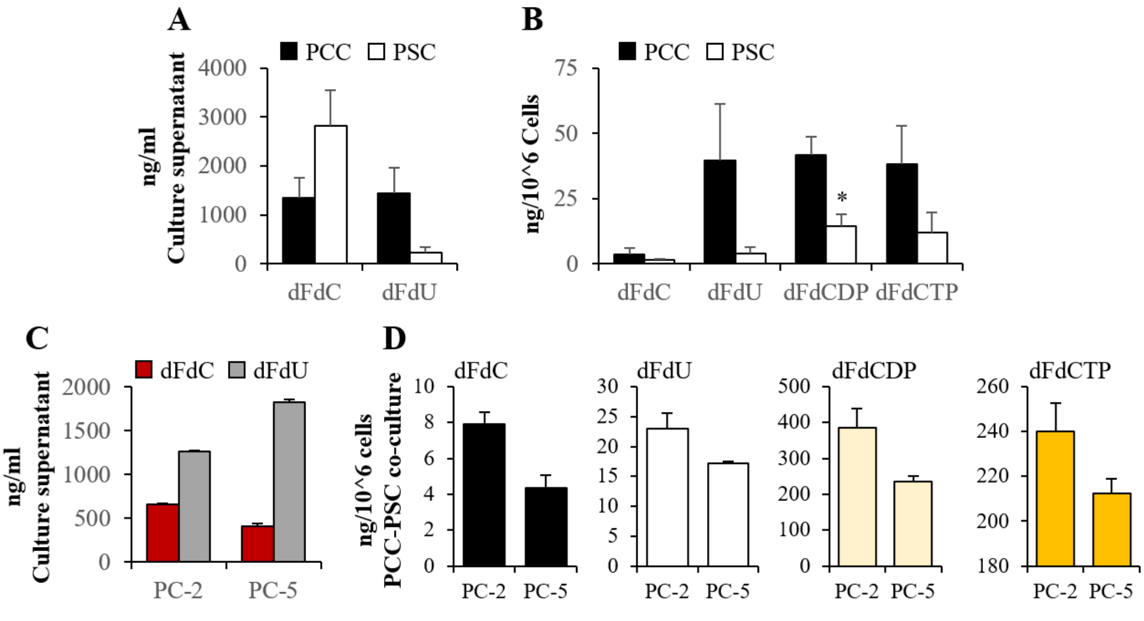

Figure S2. Pharmacokinetic profile of gemcitabine and its metabolites. Human PDAC-derived primary PCCs (n = 4) and PSCs (n = 3) were incubated with gemcitabine (10 μM) for 2 h. Cell pellets

and culture supernatants were subjected to LC-MS/MS analysis for determination of gemcitabine prodrug dFdC, its inactive form dFdU, and its metabolites (dFdCDP, dFdCTP). Data analysis comparing PCCs vs PSCs culture supernatants (**A**) and cell pellets (**B**), co-cultures of PCC-PSCs culture supernatants (**C**) and cell pellets (**D**) among culture supernatants (extracellular) and cell pellets (intracellular). Data are mean ± SEM of three replicates. LC-MS/MS, liquid chromatography tandem mass spectrometry; PCC, pancreatic cancer cell; PSC, pancreatic stellate cell.

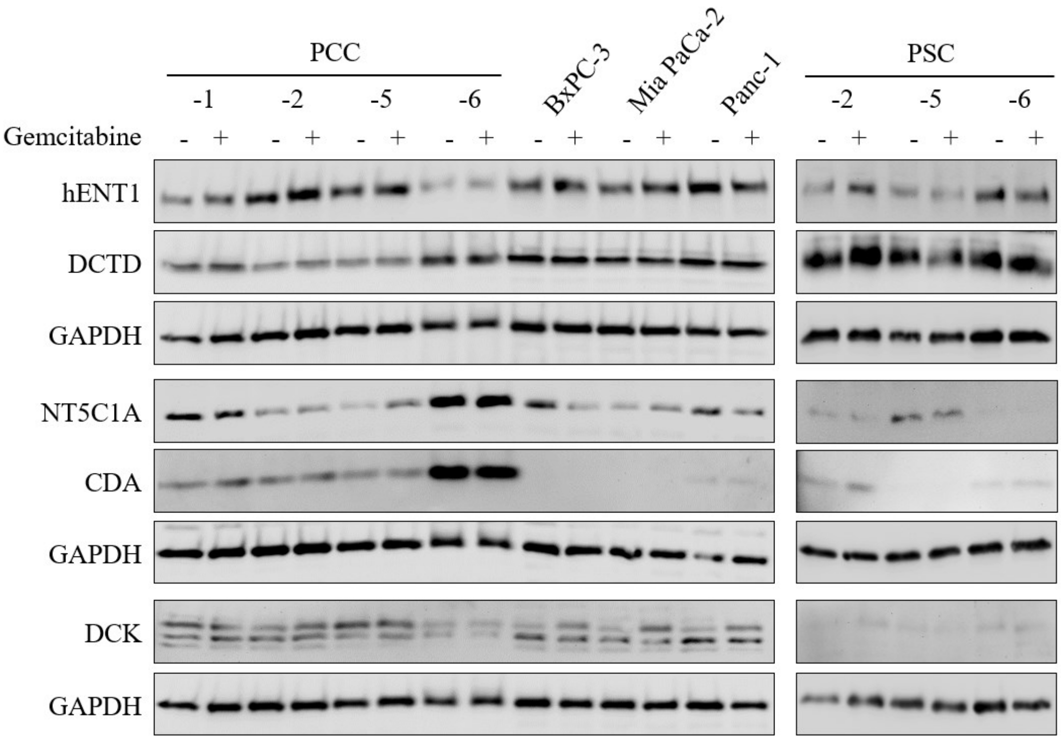

Figure S3. Expression of key regulators of intracellular gemcitabine metabolism was not influenced by gemcitabine exposure. Both PCCs and PSCs, and pancreatic cancer cell lines exposed to gemcitabine (10 μM) for 48 h were lysed and proteins were subjected to immunoblotting using antibodies against hENT1, DCTD, NT5C1A, CDA, and DCK. GAPDH was used as a loading control. PCC, pancreatic cancer cell; PSC, pancreatic stellate cell.

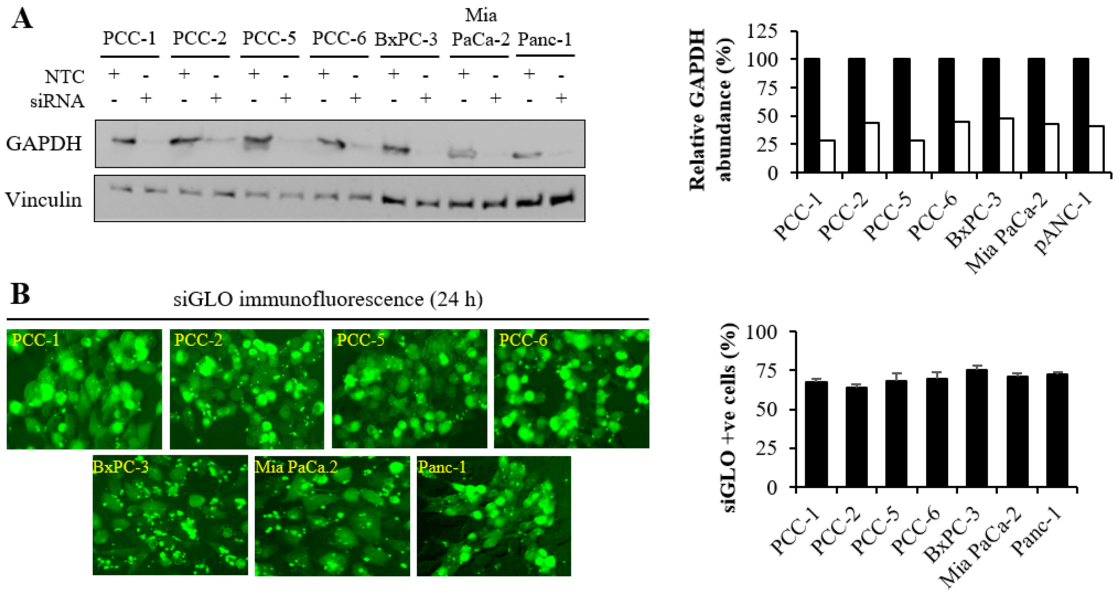

Figure S4. Transfection efficiency. (**A**) PCCs were transiently transfected siRNA against GAPDH using Lipofectamine RNAiMAX reagent (Invitrogen). Cells incubated for 72 h post-transfection were

lysed and proteins subjected to immunoblotting using antibodies against GAPDH. Vinculin were used as a loading control. (**B**) Immunofluorescence images of PCCs transfected with siGLO using Lipofectamine RNAiMAX reagent for 24 h. Percentage of cells with positive nuclear staining indicate transfection efficiency. Data are mean ± SEM. NTC, negative transfection control; PCC, pancreatic cancer cell.

Uncropped western blot images : Figure 5C

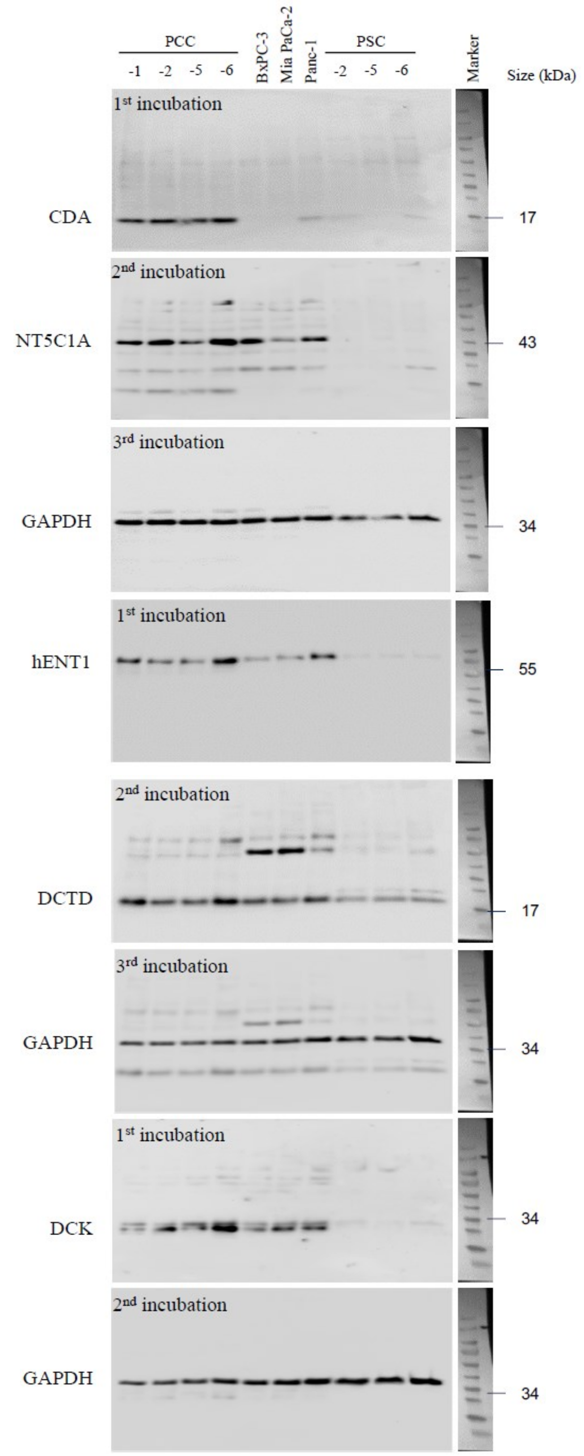

Uncropped western blot images : Figure 6A

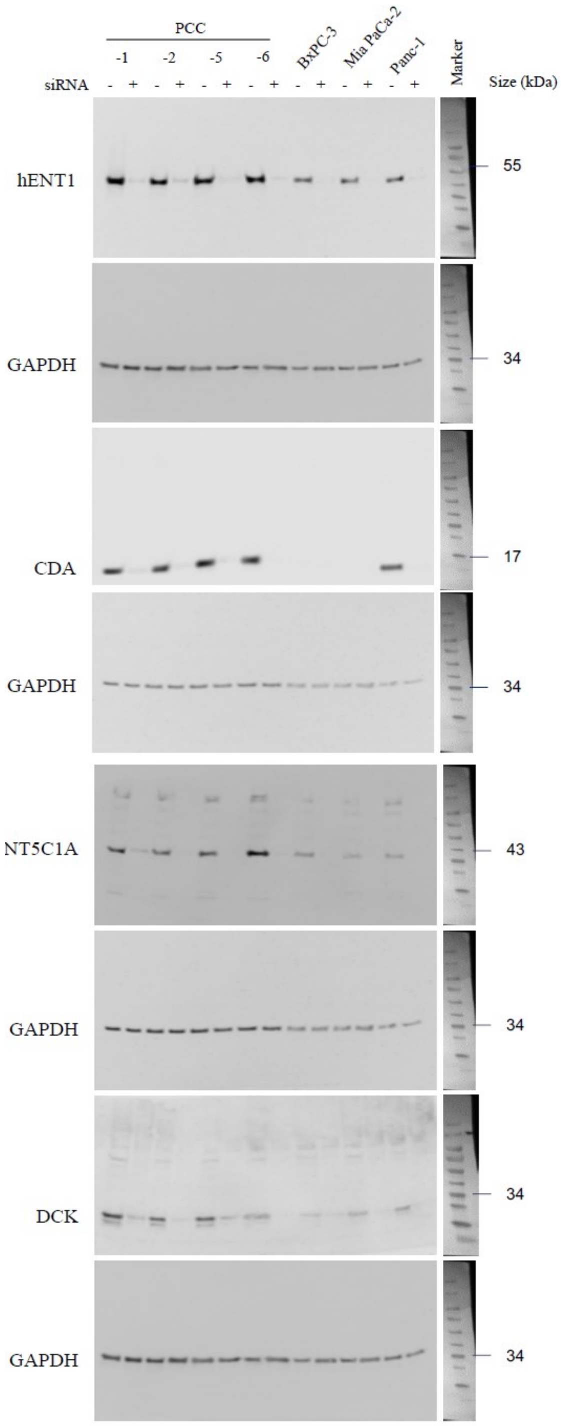

Uncropped western blot images: Figure S3

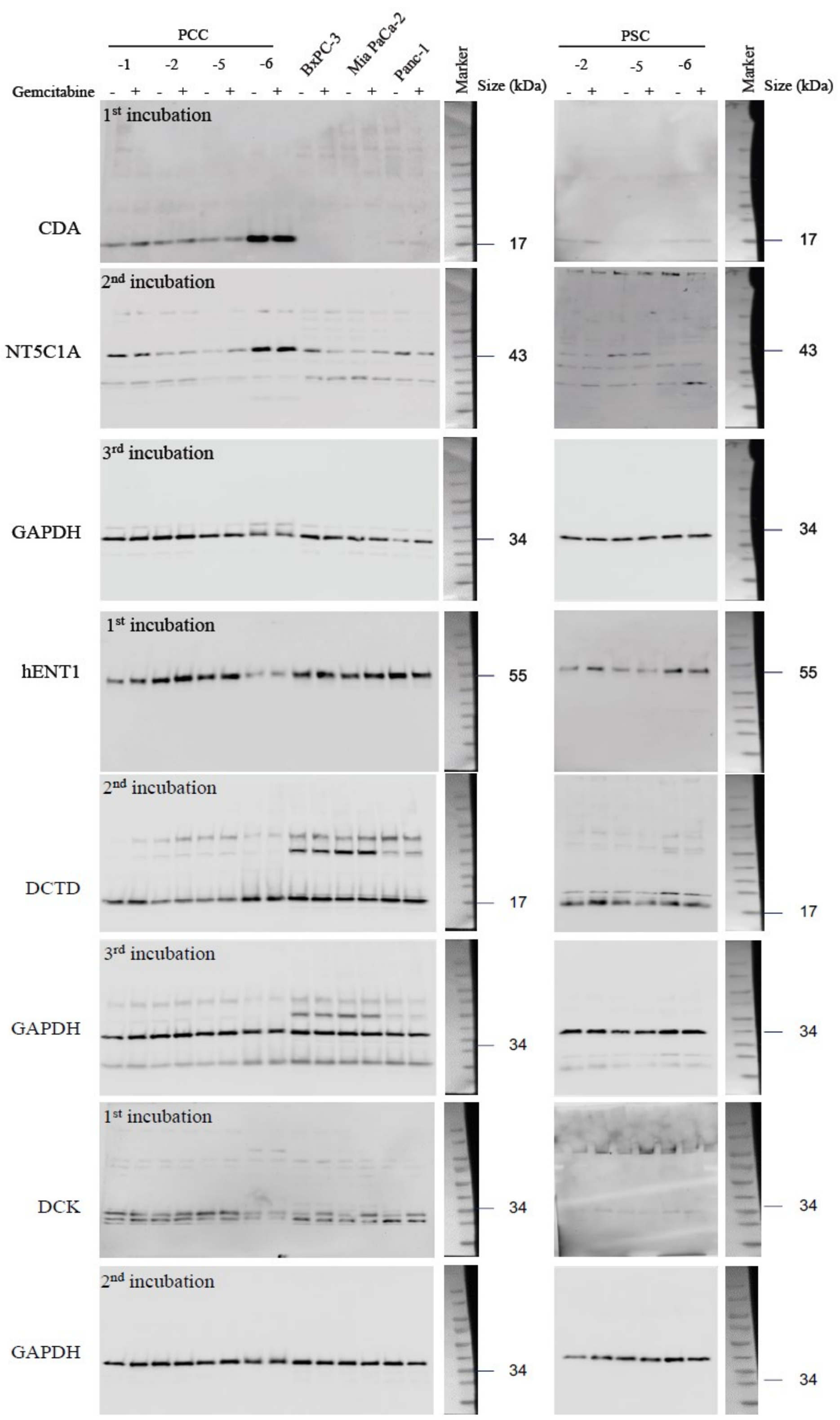

Uncropped western blot images: Figure S4A

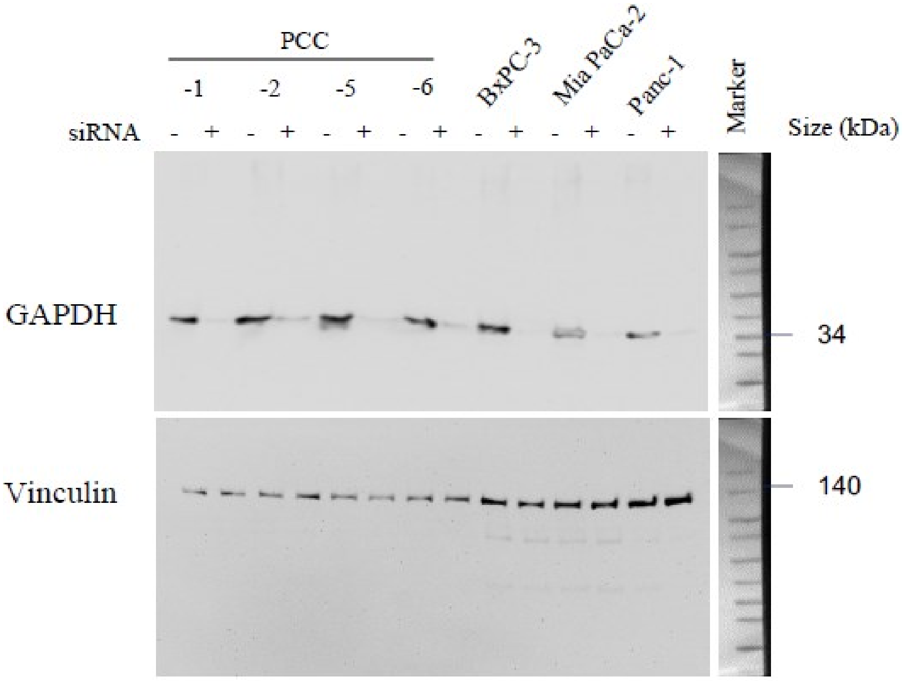

Figure S5. Uncropped western blot images.

Table S1. Antibodies details.

**Primary antibodies**				
**Name**	**Supplier**	**Cat.no. and RRID**	**Dilution**	**Application**
hENT1<br>(human equilibrative<br>nucleoside transporter -1)	Proteintech	11337-1-AP<br>(RRID: AB_2190784)	1:1000<br>1:400<br>1:50	WB<br>IHC<br>ICC
CDA<br>(cytidine deaminase)	LifeSpan Biosciences Inc.	LS-B10533<br>(RRID: not available)	1:1000<br>1:100<br>1:200	WB<br>IHC<br>ICC
DCK<br>(deoxycytidine kinase)	LifeSpan Biosciences Inc.	LS-B10837<br>(RRID: not available)	1:1000<br>1:200<br>1:200	WB<br>IHC<br>ICC
NT5C1A<br>(5′-nucleotidase cytosolic 1A)	Assay Biotech	C15296<br>(RRID: AB_10687827)	1:1000<br>1:300<br>1:200	WB<br>IHC<br>ICC
DCTD<br>(deoxycytidylate deaminase)	Elabscience	EPP12174<br>(RRID: not available)	1:1000<br>1:1000<br>1:200	WB<br>IHC<br>ICC
Cytokeratin 19	Abcam	ab52625<br>(RRID: AB_2281020)	1:200	ICC
◎-SMA	Nordic Biosite AB	BSH-7459<br>(RRID: not available)	1:200	ICC
Vimentin	Cell Signaling<br>Technology	#5741<br>(RRID: BDSC_5741)	1:200	ICC
**Secondary antibodies (IgG)**				
**Name**	**Supplier**	**Cat.no. and RRID**	**Dilution**	**Application**

Alexa Fluor 488 AffiniPure Donkey Anti-Mouse	Jackson ImmunoResearch Laboratories, Inc.	715-545-150 (RRID: AB_2340846)	1:500	ICC
Alexa Fluor 594 AffiniPure Goat Anti-Rabbit	Jackson ImmunoResearch Laboratories, Inc.	111-585-144 (RRID: AB_2307325)	1:500	ICC
HRP-Conjugated Goat Anti-Mouse	Bio-Rad Laboratories	1706516 (RRID: AB_11125547)	1:10 000	WB
HRP-Conjugated Goat Anti-Rabbit	Bio-Rad Laboratories	1706515 (RRID: AB_11125142)	1:10 000	WB
Immpress HRP Anti-Rabbit	Vector Laboratories	MP-7401 (RRID: AB_2336529)	Ready to use	IHC
Immpress HRP Anti-Mouse	Vector Laboratories	MP-7402 (RRID: AB_2336528)	Ready to use	IHC

Expression pattern

**Protein name**	**Acinar cells**	**Islets**	**Duct epithelium**	**Other**	**Suggested control**
hENT1	-<br>moderate	-<br>weak	few +<br>no info		acinar cells
CDA	-<br>-	-<br>-	-<br>-	neutrophils +<br>neutrophils + (bone marrow)	neutrophils
DCK	weak<br>-	weak<br>-	-<br>-	lymphoid cells<br>lymphoid cells (tonsil)	lymphoid cells
NT5C1A	moderate<br>weak-moderate	-<br>-/weak	-/weak<br>no info	small bowel	acinar cells
DCTD	weak<br>moderate	weak/moderate<br>moderate	-/weak<br>no info	duodenum	acinar cells

Expression in normal pancreas and according to human protein atlas are indicated in black and blue color, respectively. ICC, immunocytochemistry (immunostaining); IHC, immunohistochemistry; WB, western blot.

Table S2. siRNA details.

**Target gene**	**Supplier**	**Cat no.**	**siRNA ID**	**Target exons**
**hENT1** (SLC29A1; human equilibrative nucleoside transporter -1)	Thermo Fisher Scientific	AM51331	117291	13, 14
**CDA** (cytidine deaminase)	Thermo Fisher Scientific	AM16708A	119608	4
**DCK** (deoxycytidine kinase)	Thermo Fisher Scientific	AM51331	69	2, 3
**NT5C1A** (5′-nucleotidase cytosolic 1A)	Thermo Fisher Scientific	AM16708A	34390	4
Silencer™ Negative Control No. 1 siRNA	Thermo Fisher Scientific	AM4611		-
Silencer™ Select GAPDH Positive Control siRNA	Thermo Fisher Scientific	4390849		-

Table S3. Correlation between gemcitabine IC_{50} values and its metabolites levels or protein expression of metabolizing enzymes in pancreatic cancer cells.

**Protein expression relative to GAPDH**									
**Protein name**	PCC-1	PCC-2	PCC-5	PCC-6	BxPC-3	Mia PaCa-2	Panc-1	**Spearman's correlation coefficient**	**p-values (*p < 0.05)**
hENT1	0.79	0.51	0.44	1.09	0.18	0.19	0.43	-0.36	0.43
DCK	0.41	0.39	0.34	0.83	0.05	0.05	0.1	-0.36	0.43
CDA	0.64	1.09	0.42	2.1	0.97	0.58	0.71	-0.036	0.94
NT5C1A	0.64	0.99	0.87	1.83	0.53	0.47	0.53	-0.54	0.21
DCTD	6.68	4.22	4.24	5.69	3.57	2.56	2.36	-0.61	0.14
**Gemcitabine IC_{50} (μM)**	1.18	0.29	1.18	4.34	4.23	7.91	10.47	-	-
**Correlation between gemcitabine IC_{50} values and metabolite levels or protein expression ratio**									
**Metabolite levels** (relative to gemcitabine IC_{50} values)									
dFdCDP								-0.81	0.03*
dFdCTP								-0.81	0.03*
dFdCDP + dFdCTP								-0.87	0.01*
**Protein expression ratio** (relative to gemcitabine IC_{50} values)									
hENT1/CDA								0.86	0.01*
hENT1/NT5C1A								-0.20	0.67
hENT1/DCTD								0.15	0.74
DCK/CDA								0.45	0.31
DCK/NT5C1A								-0.67	0.10
DCK/DCTD								0.09	0.84
hENT1*DCK								-0.43	0.33
CDA*NT5C1A								-0.48	0.26
hET1/CDA*NT5C1A								0.59	0.15
DCK/CDA*NT5C1A								0.49	0.26
hENT1*DCK/CDA								0.70	0.07
hENT1*DCK/NT5C1A								-0.30	0.50
hENT1*DCK/DCTD								-0.09	0.84
hENT1*DCK/CDA*NT5C1A								0.57	0.15
hENT1*DCK/CDA*DCTD								0.85	0.02*
hENT1*DCK/NT5C1A*DCTD								-0.02	0.97
hENT1*DCK/CDA*NT5C1A*DCTD								0.58	0.17

Supplemental Methods: Quantification of Gemcitabine and Its Metabolites in Cells and Culture Supernatants

1.1. Reagents

Gemcitabine (2′,2′-difluorodeoxycytidine; dFdC), 2′,2′-difluorodeoxycytidine-5′-diphosphate (dFdCDP), 2′,2′-difluorodeoxycytidine-5′-triphosphate (dFdCTP), 2′,2′-difluorodeoxyuridine (dFdU), $^{13}C,^{15}N_2$-dFdC, and $^{13}C,^{15}N_2$-dFdU were obtained from Toronto Research Chemicals, Toronto, Canada. 2'-deoxycytidine-$^{13}C_9,^{15}N_3$-5'-triphosphate ($^{13}C_9,^{15}N_3$-dCTP), LC-MS grade formic acid, ammonium acetate and ammonium hydroxide were products of Sigma-Aldrich, St. Louis, MO, USA. Tetrahydrouridine was purchased from Merck-Millipore, Darmstadt, Germany. LC-MS grade acetonitrile was obtained from Thermo Fisher Scientific, Waltham, MA, USA and UHPLC-MS grade water and methanol were from Honeywell, NJ, USA.

1.2. Cell Treatment

Briefly, primary cultures of PCCs, PDAC cell lines BxPC-3, Mia PaCa-2, and Panc-1, and primary PSC cultures seeded in 12-well plates were cultured to confluence and treated with gemcitabine (10 μM) and incubated for 2 h. Post-incubation, cell culture supernatant was collected, centrifuged (10000

rpm for 3 min) and stored in −80 °C. Cells were washed twice with ice-cold PBS, followed by trypsinization and centrifugation. The cell pellets were washed twice with ice-cold PBS and stored in −80 °C.

1.3.1. Sample Preparation for LC-MS/MS

Cells and medium were prepared with slight modifications of the procedure described by Bapiro et al. [1]. The cell pellets in microcentrifuge tubes were kept on ice and added 200 µL ice-cold acetonitrile: water (50:50 v/v) containing tetrahydrouridine (25 mg/L) and the internal standards (^{13}C,$^{15}N_2$-dFdC 85.0 µg/L, $^{13}C_9$,$^{15}N_3$-dCTP 85.0 µg/L and ^{13}C,$^{15}N_2$-dFdU 100 µg/L). The mixture was shaken for 10 minutes (1600 rpm, 3 mm orbit) and centrifuged at 10000 g for 5 minutes at 4 °C. Thereafter, 180 µL supernatant was transferred to a glass tube and evaporated to dryness under nitrogen gas at 40 °C. The residue was reconstituted in 200 µL water and centrifuged at 1200 g for 5 minutes at 4 °C. Then, 150 µL was transferred to liquid chromatography (LC) vials and placed on the autosampler kept at 10 °C. The preparation of cell medium (25 µL sample volume) was performed with extraction solution based on acetonitrile: water 85:15 v/v. Otherwise, the procedure was the same as for cell pellets.

1.3.2. LC-MS/MS Analysis

The quantification of dFdC, dFdCDP and dFdCTP was fitted on a Transcend II LX-2 TSQ Quantiva system (Thermo Fisher Scientific, Waltham, MA) based on the LC tandem mass spectrometry (MS/MS) method reported by Bapiro et al. [1]. Briefly, a volume of 10 µL was injected on a PGC Hypercarb column 100 × 2.1 mm, 5 µm with the guard Hypercarb 10 × 2.1 mm, 5 µm in front (Thermo Fisher Scientific). Mobile phase A consisted of 10 mmol/L ammonium acetate adjusted to pH 10 with ammonium hydroxide, and mobile phase B was acetonitrile. The flow rate was 300 µL and column temperature 25 °C. The gradient was as follows: 95 % mobile phase A for 2 minutes, decrease to 80 % A for 0.2 minutes, 80% A for 5.6 minutes, increase to 95% A for 0.2 minutes, 95 % A for 7 minutes (sample run-time 15 minutes). The MS/MS was operated in positive mode with electrospray voltage 3.4 kV, capillary temperature 270 °C, and vaporizer temperature 470 °C. The sweep, sheath and auxiliary nitrogen gas flows were set at the arbitrary units 4, 60 and 8, respectively. With compound-optimized collision energy and RF lens values, the following mass transitions were monitored with dwell-time 0.030 seconds: dFdC m/z 264.1>112.2 and its internal standard ^{13}C,$^{15}N_2$-dFdC m/z 267.1>115.2; dFdCDP m/z 424.1>326.1, dFdCTP m/z 504.1>326.1 and their internal standard $^{13}C_9$,$^{15}N_3$-dCTP m/z 480.1>119.2.

The metabolite dFdU was separately quantified on the same LC-MS/MS system. A volume of 10 µL was injected on a Raptor Biphenyl column 50 × 2.1 mm, particle size 2.7 µm (Restek, Bellefonte, PA, USA) with column temperature set at 50 °C. The mobile phase, consisting of 0.50% methanol with 0.10% formic acid and 2.0 mmol/L ammonium acetate, was pumped at 500 µL/minute allowing isocratic separation (sample run-time 2 minutes). The positive electrospray and general MS conditions were similar as for the analysis of dFdC, dFdCDP and dFdCTP. Optimized mass transitions were monitored with dwell-time 0.040 seconds: dFdU m/z 265.1>113.2 and its internal standard m/z 268.1>116.2.

The software TraceFinder (Thermo Fisher Scientific) was applied for chromatographic peak smoothing, integration and calculation of concentrations. The ratio between analyte and internal standard peak areas was used as instrument response, and the calibration ranges were dFdC 2.50–2525 ng/mL, dFdCDP 2.30–2300 ng/mL, dFdCTP 2.40–2500 ng/mL, and dFdU 2.10–2130 ng/mL.

Reference

1. Bapiro, T.E.; Richards, F.M.; Goldgraben, M.A.; Olive, K.P.; Madhu, B.; Frese, K.K.; Cook, N.; Jacobetz, M.A.; Smith, D.M.; Tuveson, D.A., et al. A novel method for quantification of gemcitabine and its metabolites 2',2'-difluorodeoxyuridine and gemcitabine triphosphate in tumour tissue by LC-MS/MS: comparison with (19)F NMR spectroscopy. *Cancer Chemother. Pharmacol.* **2011**, *68*, 1243–1253, doi:10.1007/s00280-011-1613-0.
